# 

**Published:** 1910-10-01

**Authors:** 


THE MODERN MEDICAL AND INSTITUTIONAL JOURNAL.

Telegraphic Address: Ospedale, London.

Telephone Number: 2734 Gerrard.

NEW SERIES. No. 190, Vol. VIII. No. 1262, Vol. XLIX. SATURDAY, OCT. 1ST, 1910. PRICE THREEPENCE

